# Supplementary material for: Genomic analysis of the four ecologically distinct cactus host populations of Drosophila mojavensis
Source: BMC Genomics. 2019 Oct 12;20:732. doi: 10.1186/s12864-019-6097-z (PMC6790045; doi:10.1186/s12864-019-6097-z)
Supplement: Supplementary file 2 — Additional file 2. This file includes the supplementary Tables S2-S10 and Figures S1-S22. [file 12864_2019_6097_MOESM2_ESM.pdf]

Table S2 Pairwise Tukey HSD of $\log_2 \omega$ between Muller elements

Element	Element	Difference	Std Err Dif	Lower CL	Upper CL	*P* Value
F	C	1.078745	0.1890123	0.539991	1.617498	<.0001
F	D	1.033424	0.1892502	0.493993	1.572856	<.0001
F	A	1.014120	0.1906562	0.470681	1.557560	<.0001
F	B	0.714138	0.1892686	0.174654	1.253622	0.0022
F	E	0.667516	0.1883892	0.130538	1.204493	0.0053
E	C	0.411229	0.0480779	0.274189	0.548268	<.0001
E	D	0.365908	0.0490047	0.226227	0.505590	<.0001
B	C	0.364607	0.0514158	0.218053	0.511160	<.0001
E	A	0.346605	0.0541814	0.192168	0.501041	<.0001
B	D	0.319287	0.0522834	0.170260	0.468313	<.0001
B	A	0.299983	0.0571640	0.137045	0.462921	<.0001
A	C	0.064624	0.0563096	-0.095878	0.225127	0.8613
E	B	0.046622	0.0490758	-0.093262	0.186506	0.9333
D	C	0.045320	0.0513479	-0.101040	0.191681	0.9508
A	D	0.019304	0.0571029	-0.143460	0.182068	0.9994

Table S3 Pairwise Tukey HSD of $\log_2 \omega$ between different coding gene length bins.

Length Bin	Length Bin	Difference	Std Err Dif	Lower CL	Upper CL	*P* Value
1	4	1.187473	0.0668196	1.00516	1.369784	<.0001
1	5	1.147404	0.0627699	0.97614	1.318665	<.0001
1	3	1.051908	0.0494634	0.91695	1.186864	<.0001
1	2	0.767654	0.0384280	0.66281	0.872501	<.0001
2	4	0.419819	0.0648992	0.24275	0.596890	<.0001
2	5	0.379750	0.0607215	0.21408	0.545422	<.0001
2	3	0.284254	0.0468366	0.15647	0.412043	<.0001
3	4	0.135565	0.0719849	-0.06084	0.331968	0.3264
3	5	0.095496	0.0682424	-0.09070	0.281688	0.6281
5	4	0.040069	0.0817022	-0.18285	0.262985	0.9883

Bin 1 = < 1 Kb, Bin 2 = 1-2 Kb, Bin 3 = 2-3 Kb, Bin 4 = 3-4 Kb, Bin 5 >4 Kb.

Table S4 Pairwise Tukey HSD of $\log_2 \omega$ between different exon number bins.

Exon Bin	Exon Bin	Difference	Std Err Dif	Lower CL	Upper CL	*P* Value
2	7	0.9818659	0.0507210	0.832289	1.131443	<.0001
1	7	0.7994915	0.0544689	0.638862	0.960121	<.0001
3	7	0.7401690	0.0529489	0.584022	0.896316	<.0001
2	5	0.6570861	0.0652903	0.464544	0.849628	<.0001
2	6	0.6087085	0.0717851	0.397013	0.820404	<.0001
2	4	0.5297340	0.0572028	0.361042	0.698426	<.0001
1	5	0.4747116	0.0682426	0.273463	0.675960	<.0001
4	7	0.4521319	0.0569040	0.284321	0.619943	<.0001
1	6	0.4263340	0.0744805	0.206690	0.645978	<.0001
3	5	0.4153892	0.0670357	0.217700	0.613079	<.0001
6	7	0.3731574	0.0715473	0.162163	0.584151	<.0001
3	6	0.3670116	0.0733762	0.150624	0.583399	<.0001
1	4	0.3473595	0.0605508	0.168794	0.525925	<.0001
5	7	0.3247798	0.0650287	0.133009	0.516550	<.0001
3	4	0.2880371	0.0591872	0.113493	0.462581	<.0001
2	3	0.2416969	0.0532698	0.084603	0.398791	0.0001
2	1	0.1823745	0.0547809	0.020825	0.343924	0.0153
4	5	0.1273521	0.0702016	-0.079674	0.334378	0.5384
4	6	0.0789745	0.0762795	-0.145975	0.303924	0.9459
1	3	0.0593224	0.0568499	-0.108329	0.226974	0.9439
6	5	0.0483776	0.0825179	-0.194969	0.291724	0.9972

Bin 1 = 1 exon, Bin 2 = 2 exons, Bin 3 = 3 exons, Bin 4 = 4 exons, Bin 5 = 5 exons, Bin 6 = 6 exons, Bin 7 > 7 exons.

Table S5 Pairwise Tukey HSD of $\log_2 \omega$ between different expression categories.

Category	Category	Difference	Std Err Dif	Lower CL	Upper CL	*P* Value
Male bias	Female bias	0.5461726	0.0494397	0.4302817	0.6620635	<.0001
Male bias	Unbiased	0.3344762	0.0397754	0.2412392	0.4277131	<.0001
Unbiased	Female bias	0.2116964	0.0425320	0.1119977	0.3113952	<.0001

Table S6 Pairwise Tukey HSD of $\log_2 \omega$ between different expression categories.

Category	Category	Difference	Std Err Dif	Lower CL	Upper CL	*P* Value
1	2	0.5368926	0.0386429	0.446310	0.6274750	<.0001
1	3	0.4194892	0.0698346	0.255791	0.5831875	<.0001
3	2	0.1174035	0.0643864	-0.033524	0.2683308	0.1620

RPKM Category 1 = <-2,2.4>, RPKM Category 2 = <2.4,6.8>, RPKM Category 3 = <6.8,11.2>

Table S7 ANOVA results of $\log_2 \omega$ differences between loci differentially expressed as a result of cactus rearing and loci differentially expressed across populations.

Source	DF	Sum of Squares	Mean Square	F Ratio	Prob > F
Cactus Effect	1	135.11	135.11	60.64	<0.0001
Error	8317	18531.41	2.23		
Total	8318	18666.52			
Population Effect	1	116.64	116.64	52.31	<0.0001
Error	8319	18549.44	2.23		
Total	8320	18666.08			

PAML significant loci removed. Cactus effect analysis composed of 8319 loci, population effect analysis composed of 8321 loci.

Table S8 List of overrepresented GO terms among databases

Gene dataset	Term dataset	Term	*P* value [a]
TOP10	Molecular process	Serine-type peptidase activity	0.0000011
		Endopeptidase activity	0.012
		Serine-type endopeptidase activity	0.0000011
		tRNA-specific ribonuclease activity	0.0074
TOP10	KEGG	Oxidative phosphorylation	0.01
PAML-FDR	Biological function	DNA metabolic process	

[a] *P* values are FDR corrected

Table S9 Karyotype of the three *D. mojavensis* lines sequenced and of the template genome line.

	Muller E (2nd chromosome)	Muller B (3rd chromosome)
Catalina Island (15081-1352.00)[a]	st/st	f^2/f^2
Mojave (MJANZA 402-8)[b]	st/st	st/st
Baja California (MJBC 155)[b]	q^5/q^5	st/st
Sonora (MJ 122)[b]	q^5/q^5	f^2/f^2

[a] Karyotype data from [1]. [b] Data from visual inspection of polytene chromosome squashes and *de novo* assembly of genomes (unpublished data, Matzkin).

Table S10 List of reference genome scaffolds used for template assembly.

D. mojavensis chromosome	Scaffold ID	Homologous *D. melanogaster* arm	Size (bp)
Muller A Chromosome 1	1786	X	4,599
	6308	X	3,356,042
	6314	X	47,931
	6328	X	4,453,435
	6359	X	4,525,533
	6473	X	16,943,266
	6482	X	2,735,782
	6541	X	2,543,558
	6657	X	3,686
	6660	X	7,398
		Total Size	34,621,230
Muller B Chromosome 3	587	2L	1,541
	6005	2L	1,209
	6200	2L	24,743
	6500	2L	32,352,404
	6583	2L	2,108
	6584	2L	818
	6587	2L	1,717
	6615	2L	6,841
	6646	2L	1,733
		Total Size	32,393,114
Muller C Chromosome 5	1740	2R	7,530
	3877	2R	1,968
	6071	2R	80,338
	6496	2R	26,866,924
		Total Size	26,956,760
Muller D Chromosome 4	4729	3L	8,237
	4734	3L	13,141
	4739	3L	8,224
	5947	3L	5,469
	5978	3L	5,259
	6654	3L	2,564,135
	6680	3L	24,764,193
		Total Size	27,368,658
Muller E Chromosome 2	1710	3R	6,556
	1718	3R	5,511
	4072	3R	13,853
	4889	3R	3,248
	6111	3R	58,398
	6368	3R	49,292
	6540	3R	34,148,556
		Total Size	34,285,414
Muller F Chromosome 6	6498	4	3,408,170
		Total Size	3,408,170
Unmapped	6499		421,677

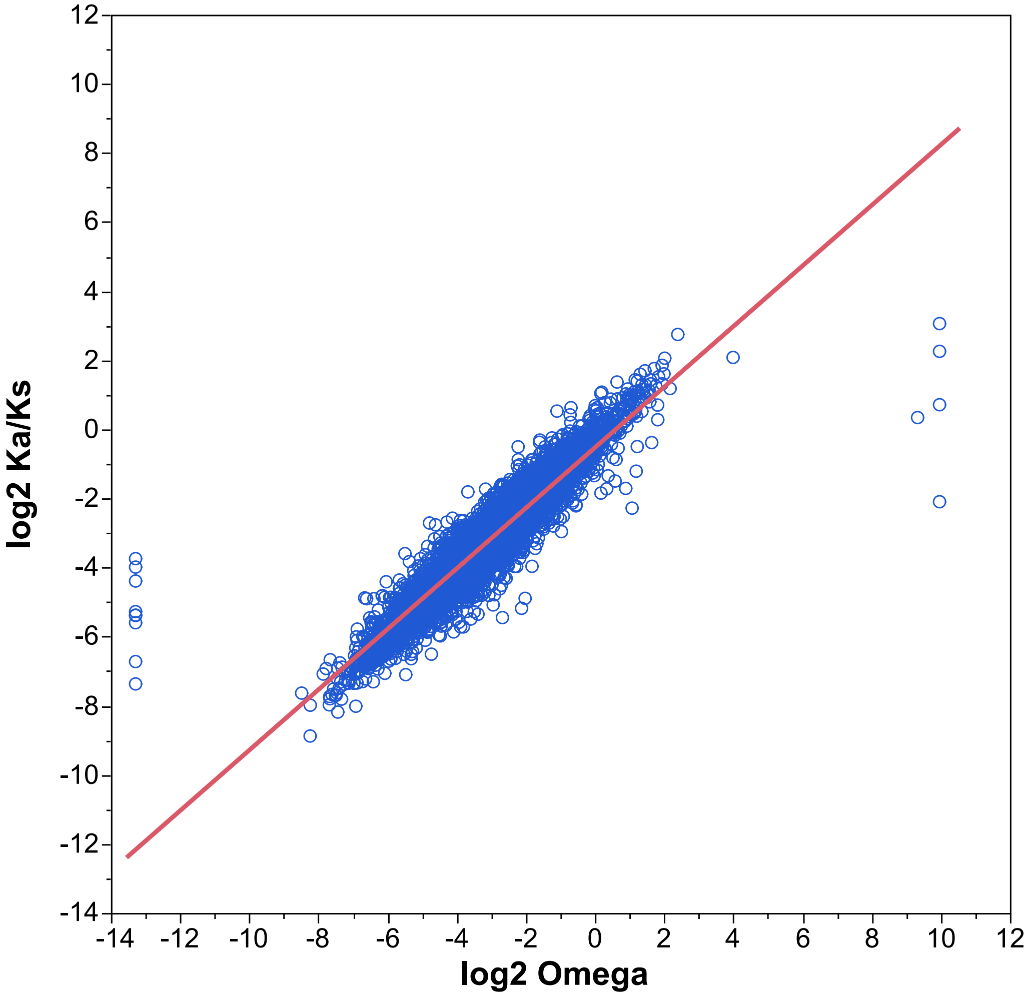

Fig. S1 Relationship between $\log_2$ Ka/Ks values estimated via the KaKs Calculator and $\log_2$ ω values estimated via Codeml among the 9,087 post filter genes ($r^2 = 0.88$, $P < 0.001$).

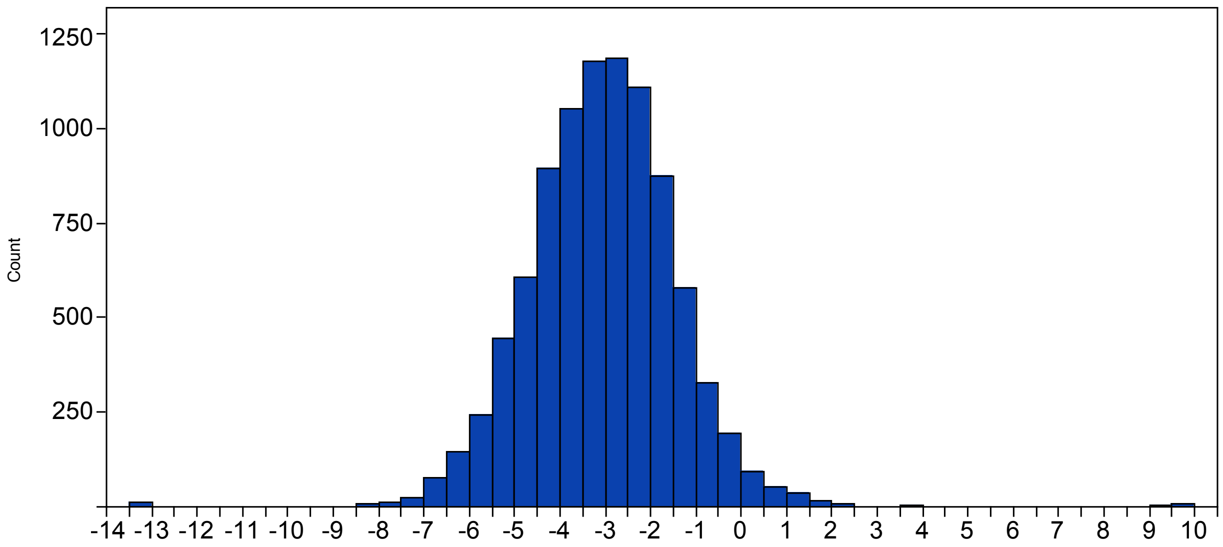

Fig. S2 Distribution of $\log_2 \omega$ values for all loci that passed filtering (>5 total substitutions, > 0 nonsynonymous substitutions, > 0 synonymous substitutions), a total of 9,087 genes.

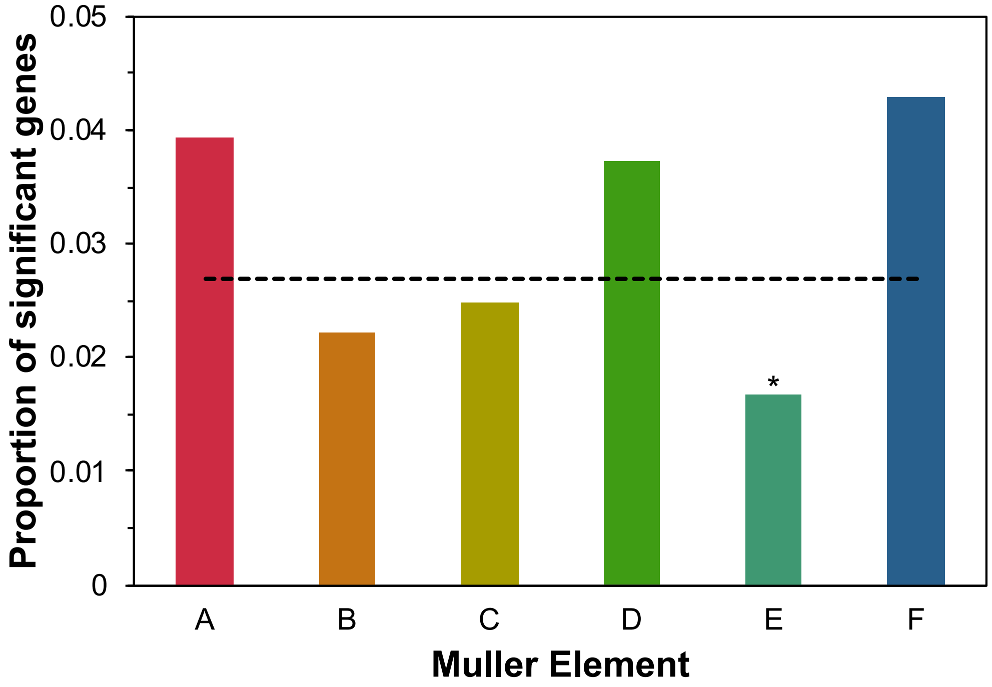

Fig. S3 Proportion of positive selected (via PAML with FDR correction) loci located in each of the *D. mojavensis* Muller elements. Dashed line indicates the genome wide proportion of positive selected loci (0.0269). Asterisk indicate significance via Fisher's Exact test (* P < 0.05, ** P < 0.01, *** P < 0.001).

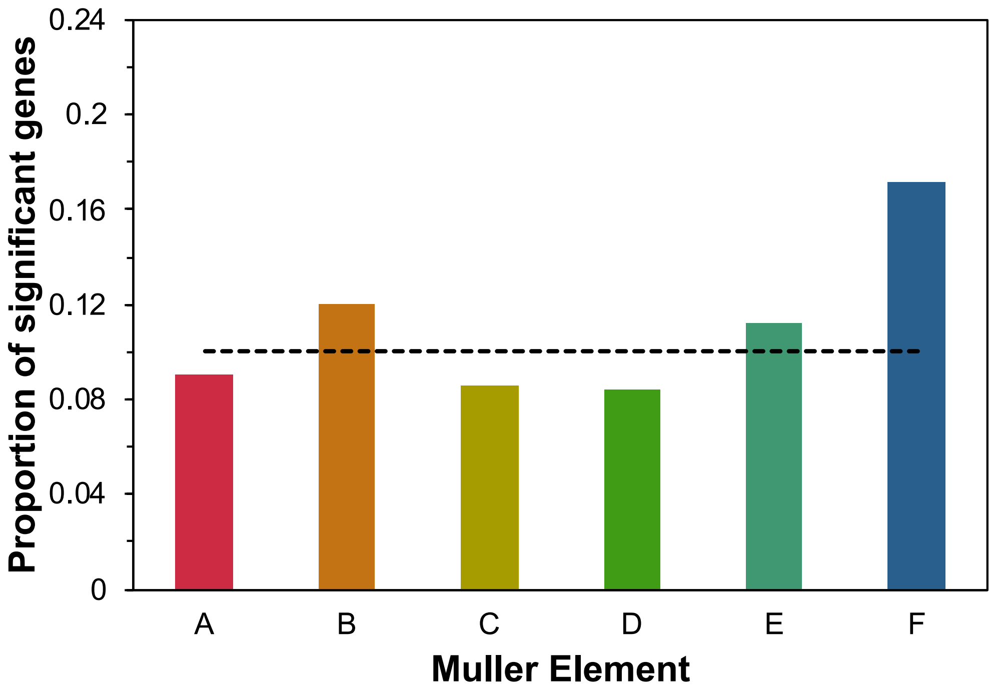

Fig. S4 Proportion of TOP10 loci located in each of the *D. mojavensis* Muller elements. Dashed line indicates the genome wide proportion of TOP10 loci (0.10). Asterisk indicate significance via Fisher's Exact test (* P < 0.05, ** P < 0.01, *** P < 0.001).

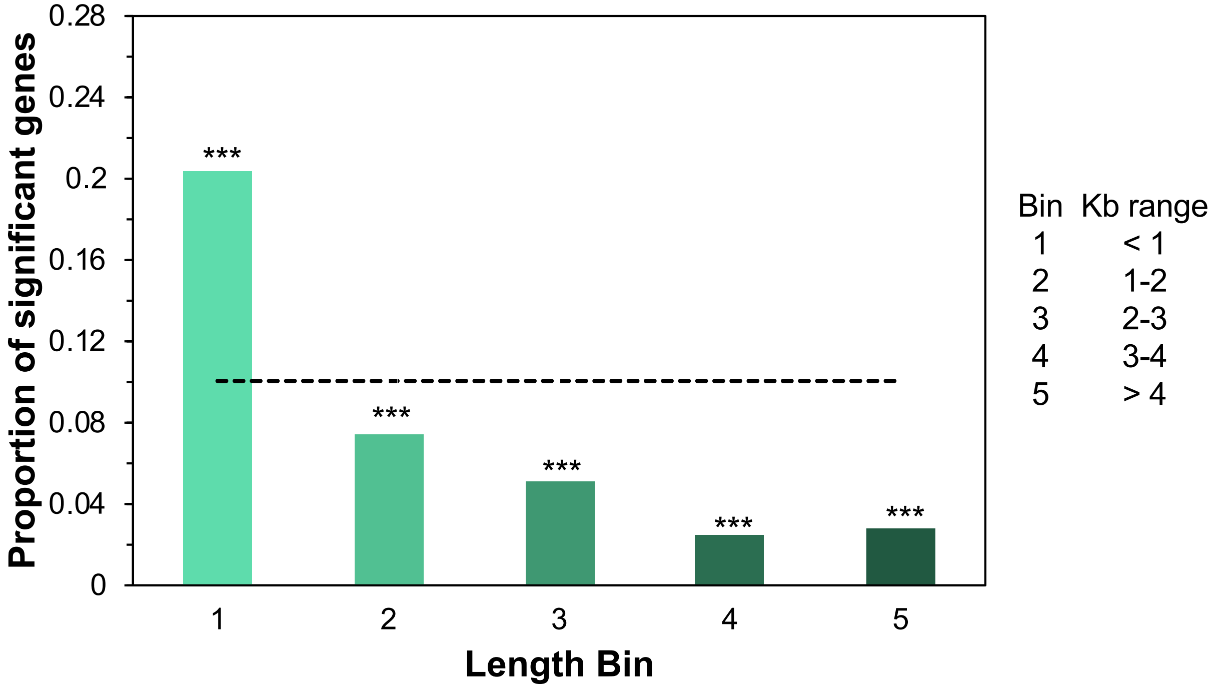

Fig. S5 Proportion of TOP10 loci in each of five coding length bins. Dashed line indicates the genome wide proportion of TOP10 loci (0.10). Asterisk indicate significance via Fisher's Exact test (* P < 0.05, ** P < 0.01, *** P < 0.001).

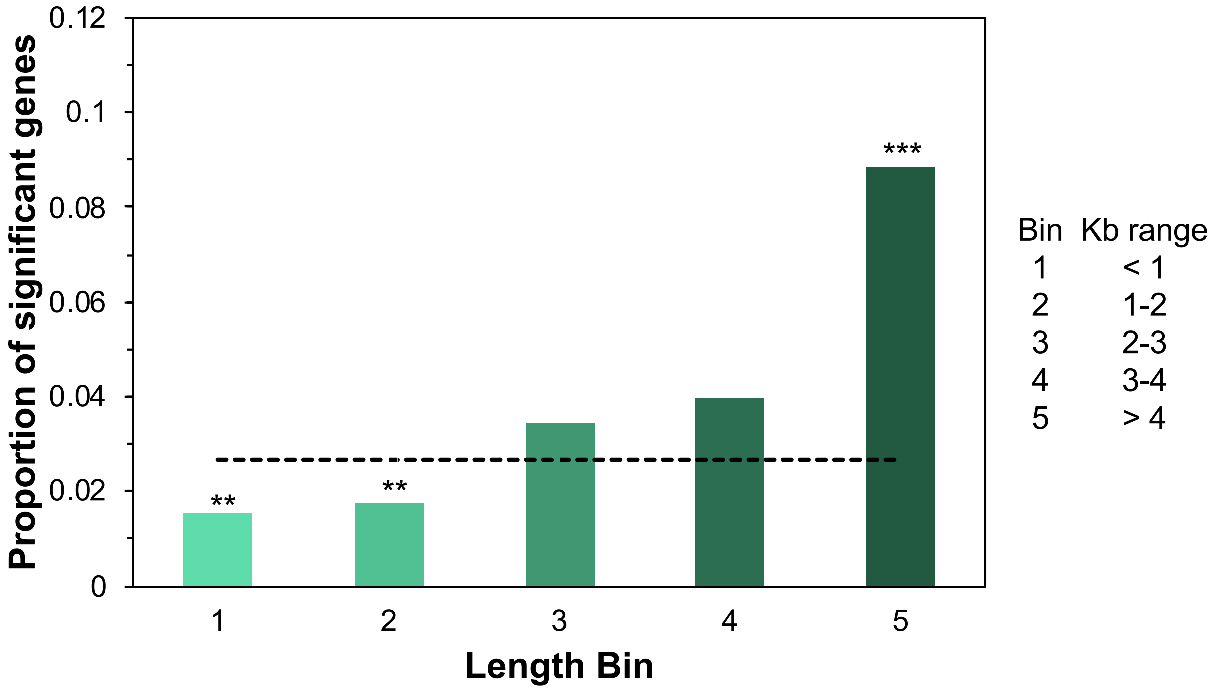

Fig. S6 Proportion of positive selected (via PAML with FDR correction) loci in each of five coding length bins. Dashed line indicates the genome wide proportion of positive selected loci (0.0269). Asterisk indicate significance via Fisher's Exact test (* P < 0.05, ** P < 0.01, *** P < 0.001).

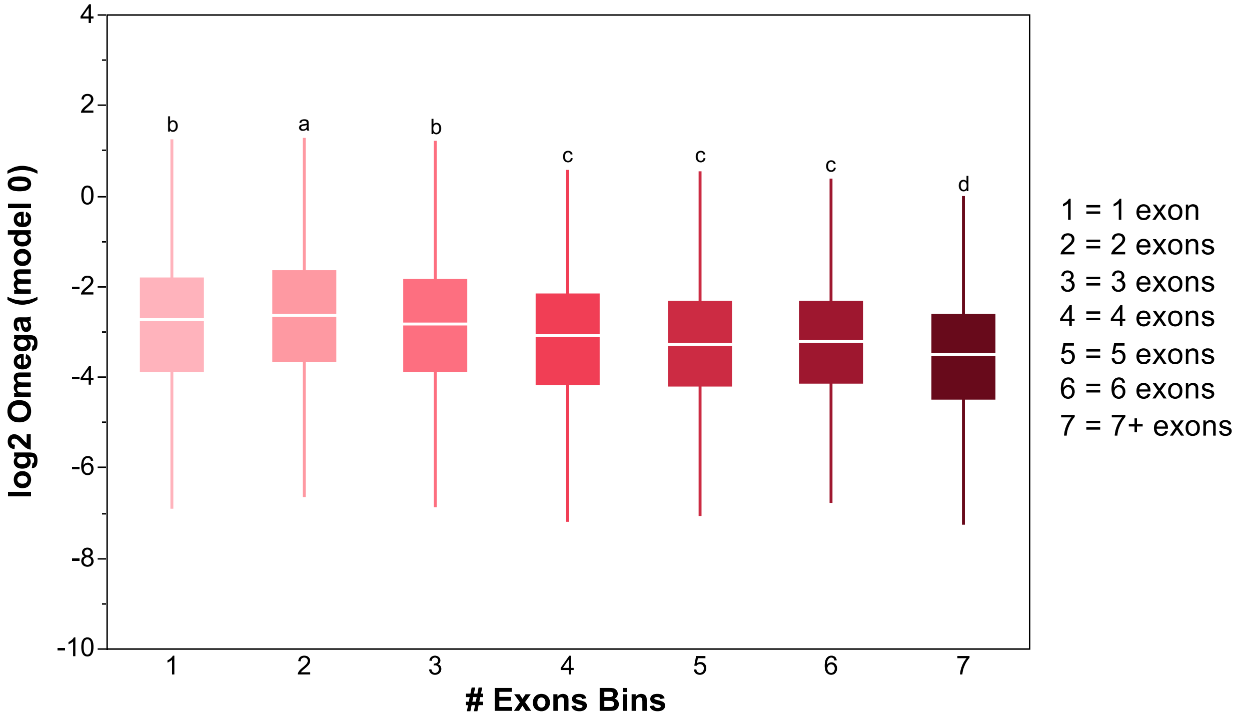

Fig. S7 Boxplot of $\log_2 \omega$ values for loci in seven different exon number bins. Bins with different letters are significantly different using a Tukey HSD test (see Table S4).

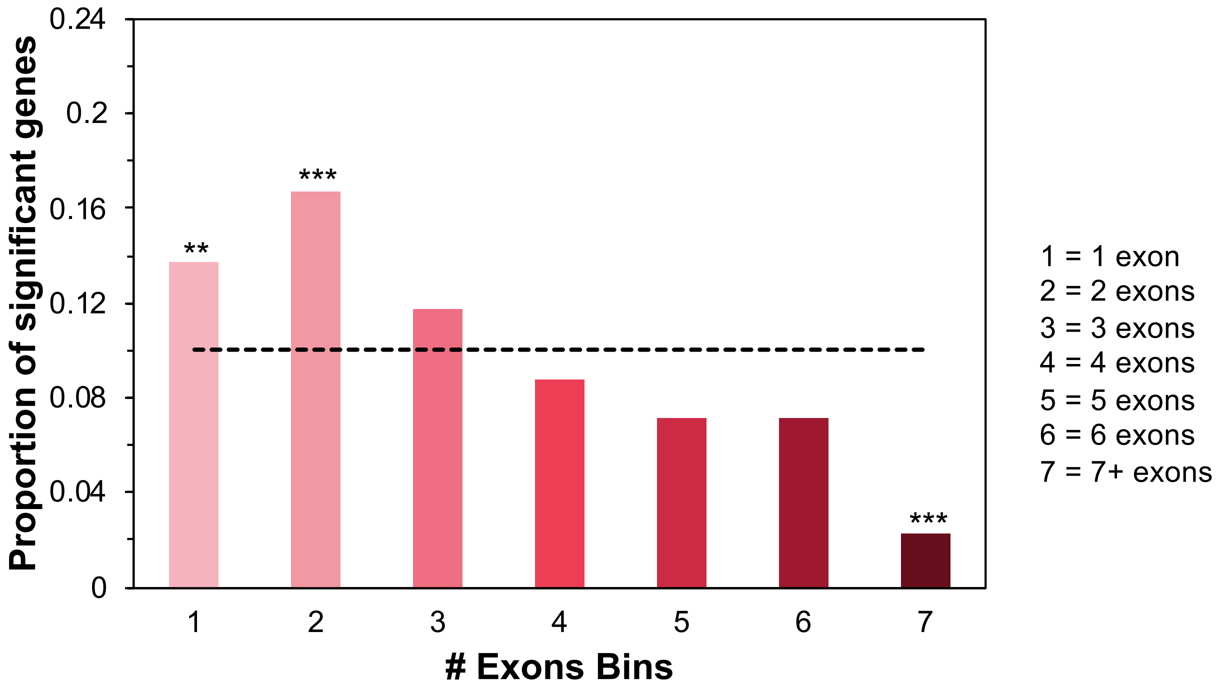

Fig. S8 Proportion of TOP10 loci in each of seven exon number bins. Dashed line indicates the genome wide proportion of TOP10 loci (0.10). Asterisk indicate significance via Fisher's Exact test (* $P < 0.05$, ** $P < 0.01$, *** $P < 0.001$).

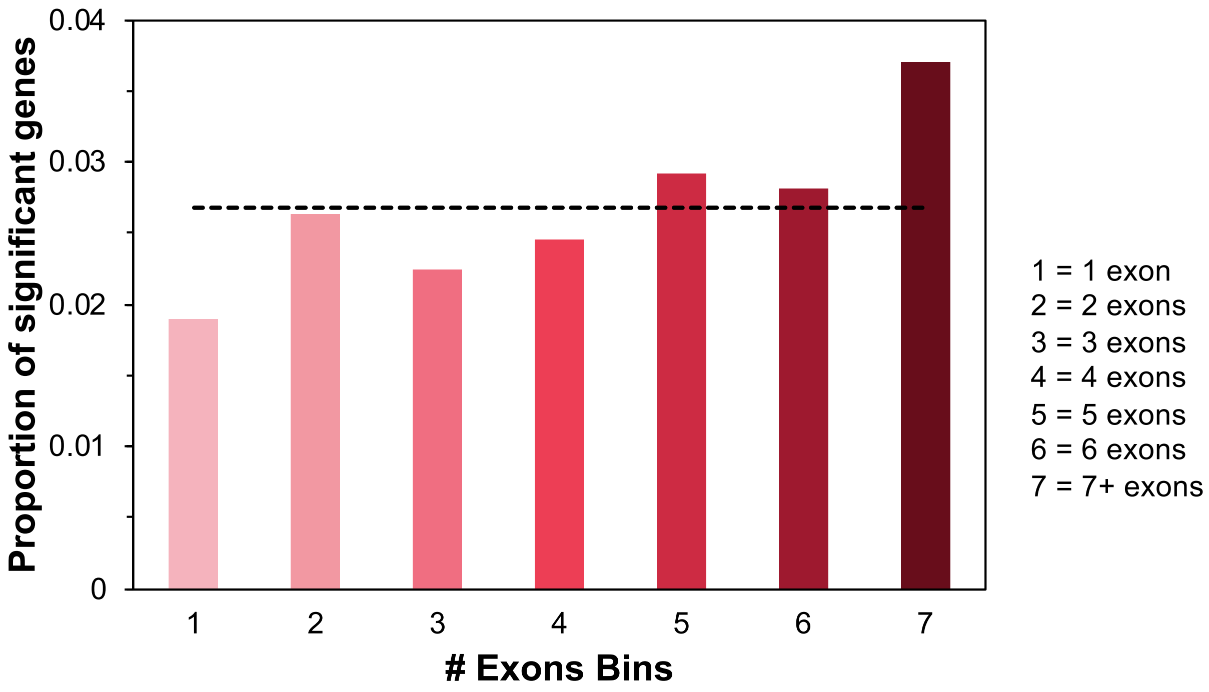

Fig. S9 Proportion of positive selected (via PAML with FDR correction) loci in each of seven exon number bins. Dashed line indicates the genome wide proportion of positive selected loci (0.0269). Asterisk indicate significance via Fisher's Exact test (* P < 0.05, ** P < 0.01, *** P < 0.001).

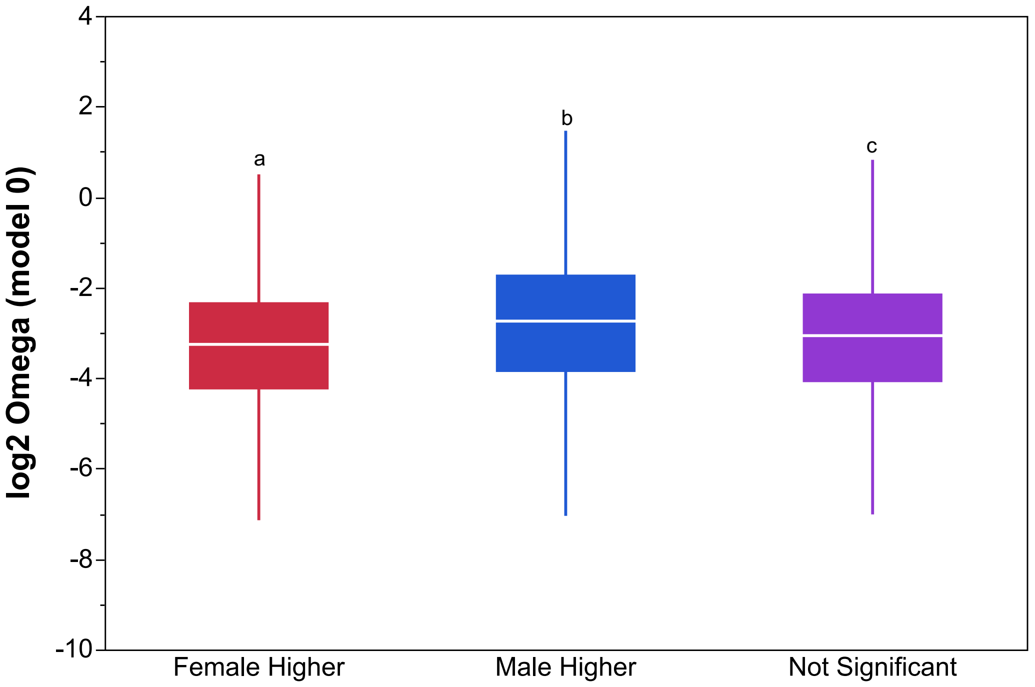

Fig. S10 Boxplot of $\log_2$ ω values for loci that show female-bias, male-bias or unbiased gene expression. Classes with different letters are significantly different using a Tukey HSD test (see Table S5). Gene expression data is from [2].

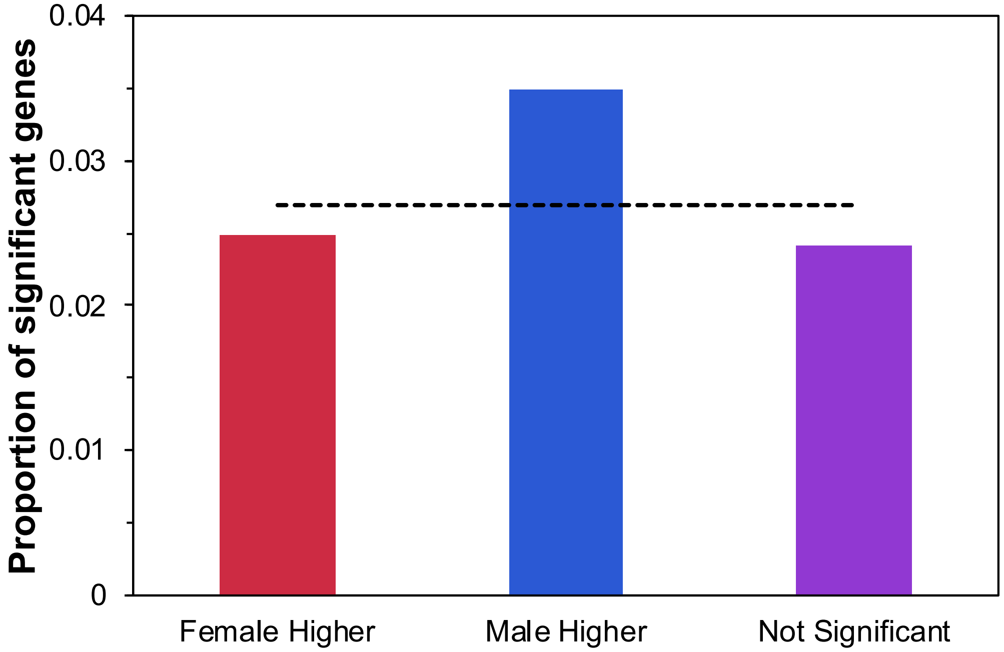

Fig. S11 Proportion of positive selected (via PAML with FDR correction) loci that show female-bias, male-bias or unbiased gene expression. Dashed line indicates the genome wide proportion of positive selected loci (0.0269). Gene expression data is from [2]. Asterisk indicate significance via Fisher's Exact test (* P < 0.05, ** P < 0.01, *** P < 0.001).

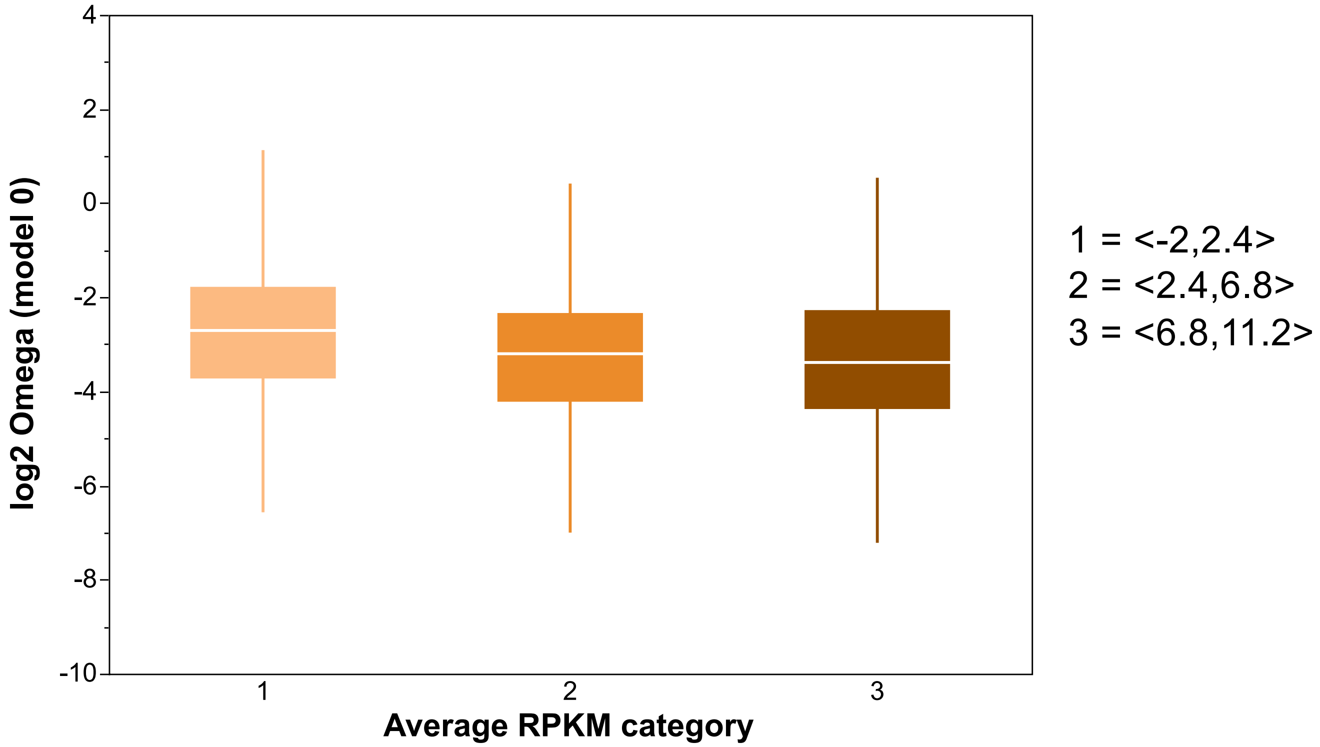

Fig. S12 Boxplot of $\log_2 \omega$ values for loci in three different expression level categories. Categories with different letters are significantly different using a Tukey HSD test (see Table S6). Gene expression data is from [2]. Female and male biased genes were removed, only 5,101 genes.

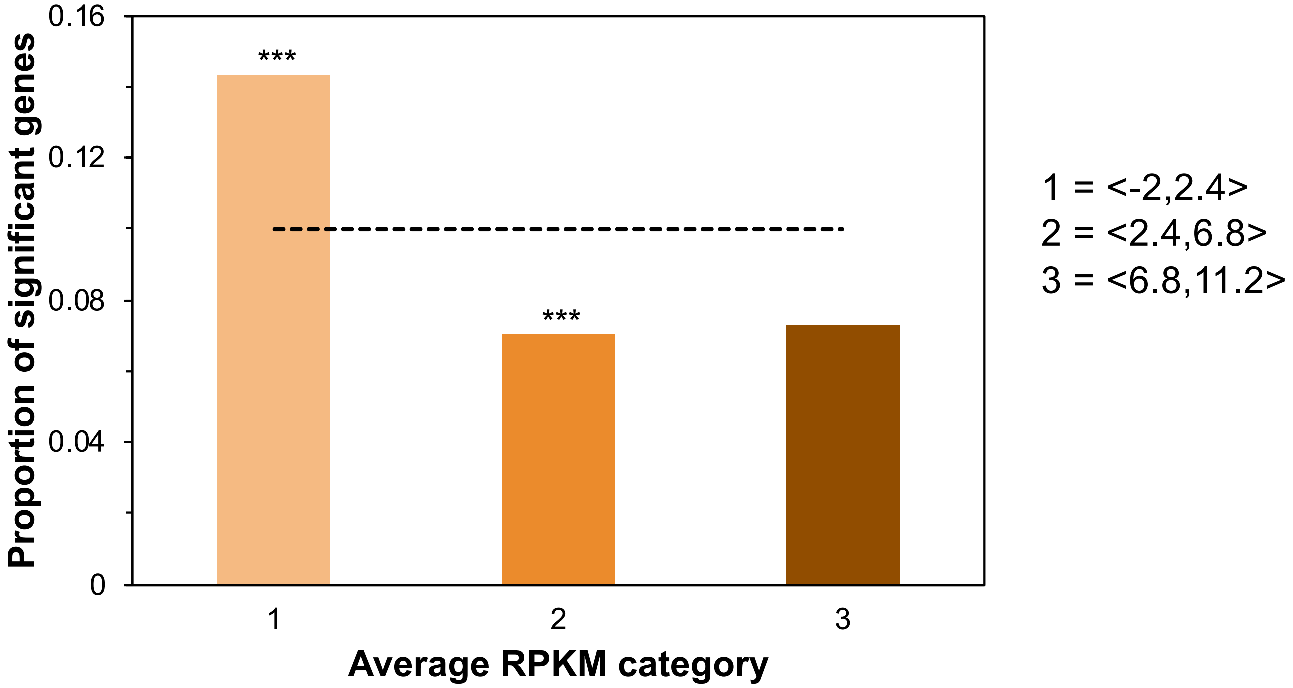

Fig. S13 Proportion of TOP10 loci in three different expression level categories. Dashed line indicates the genome wide proportion of TOP10 loci (0.10). Gene expression data is from [2]. Female and male biased genes were removed, only 5,101 genes. Asterisk indicate significance via Fisher's Exact test (* P < 0.05, ** P < 0.01, *** P < 0.001).

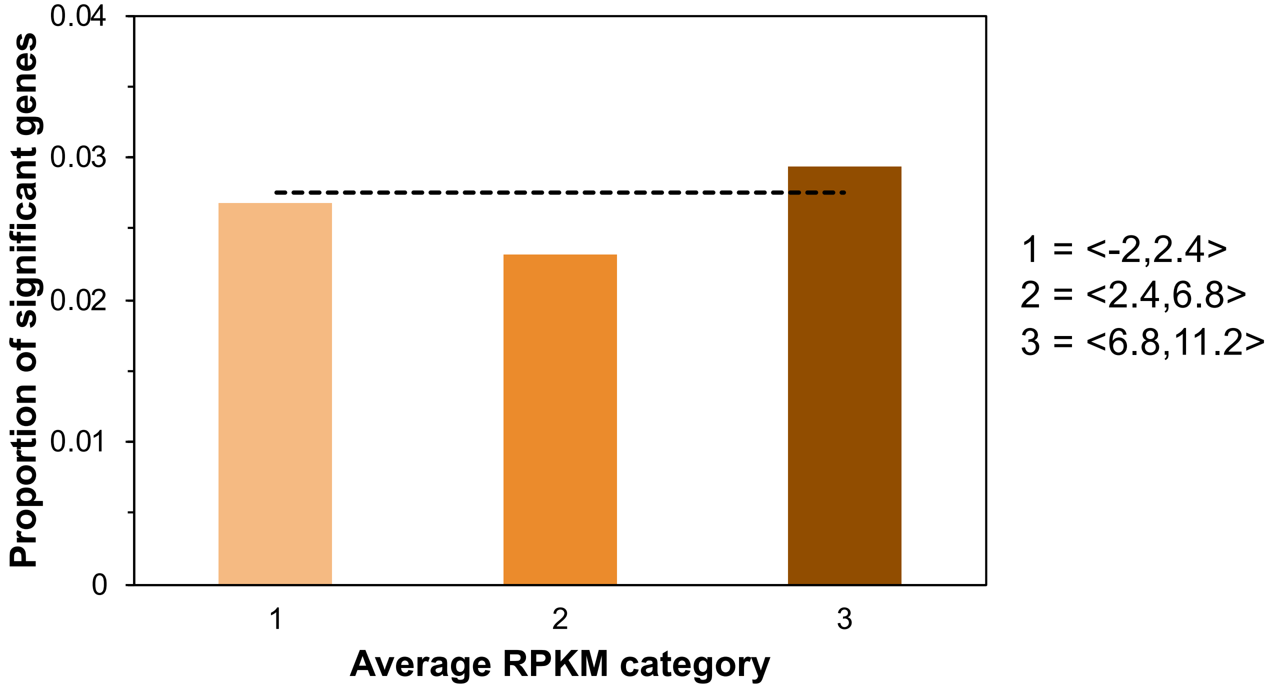

Fig. S14 Proportion of positive selected (via PAML with FDR correction) loci in three different expression level categories. Dashed line indicates the genome wide proportion of positive selected loci (0.0269). Gene expression data is from [2]. Female and male biased genes were removed, only 5,101 genes. Asterisk indicate significance via Fisher's Exact test (* P < 0.05, ** P < 0.01, *** P < 0.001).

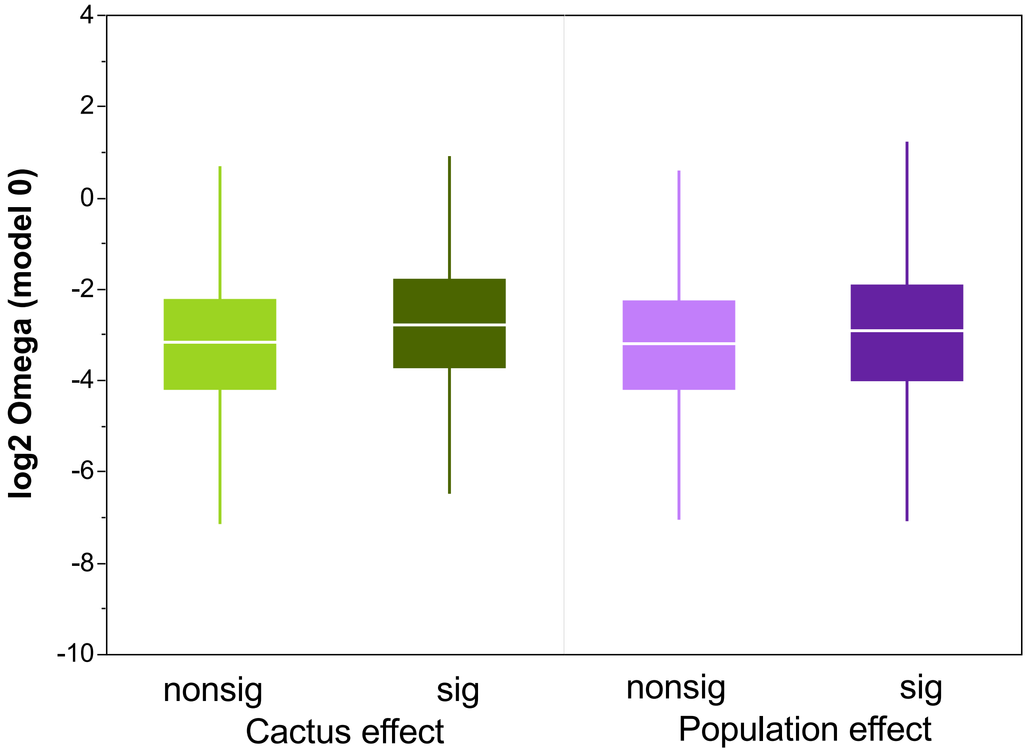

Fig. S15 Boxplot of $\log_2$ ω values for loci differentially expressed as a result of cactus rearing (left) and loci differentially expressed across populations (right). For both panels differences in $\log_2$ ω values between differentially and not differentially expressed loci were significant (ANOVA, $P < 0.001$ see Table S7). For both datasets [3, 4] only loci that had a $P < 0.001$ post FDR correction were labeled as differentially expressed. For the integrated analysis PAML significant loci were removed. Cactus effect analysis composed of 8,319 loci, population effect analysis composed of 8,321 loci.

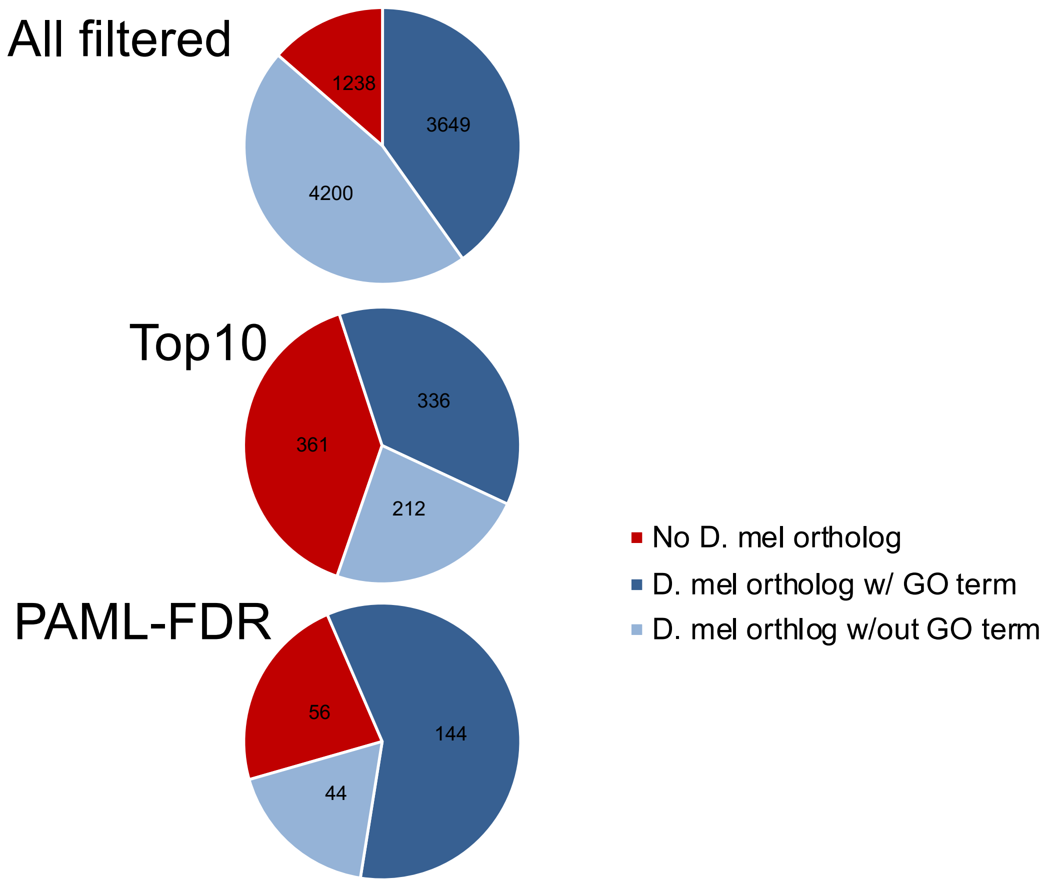

Fig. S16 Number of orthologous gene calls to the *D. melanogaster* reference genome for the filtered (9,087 genes), TOP10 (909 genes) and PAML-FDR (244 genes).

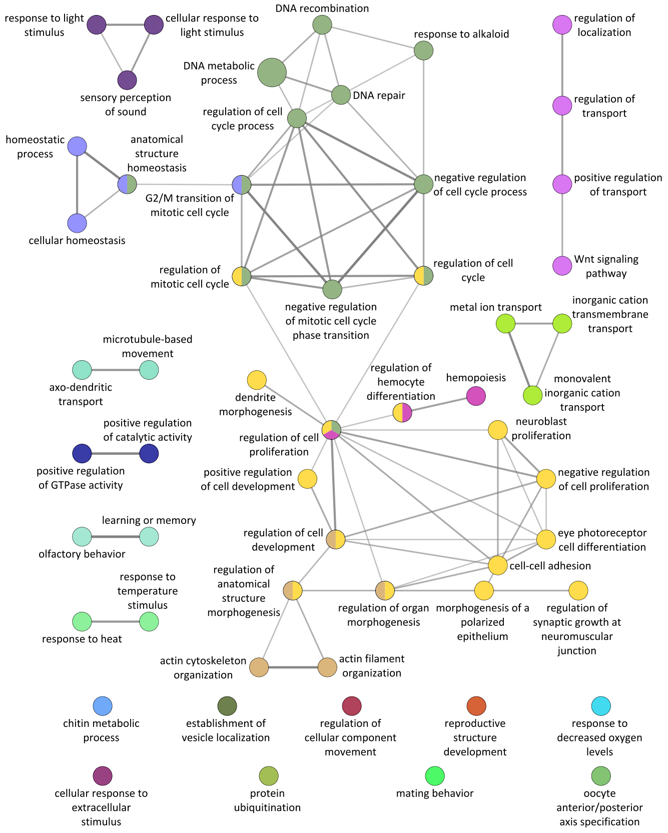

Fig. S17 Network clustering of Biological Process terms of the positive selected (via PAML with FDR correction) loci. Network clustering was performed using ClueGo using the following parameters: Min GO Level = 3, Max GO Level = 8, All GO Levels = false, Number of Genes = 10, Get All Genes = false, Min Percentage = 4.0, Get All Percentage = false, GO Fusion = true, GO Group = true, Kappa Score Threshold = 0.4 ,Over View Term = SmallestPValue, Group By Kappa Statistics = true, Initial Group Size = 1, Sharing Group Percentage = 50.0

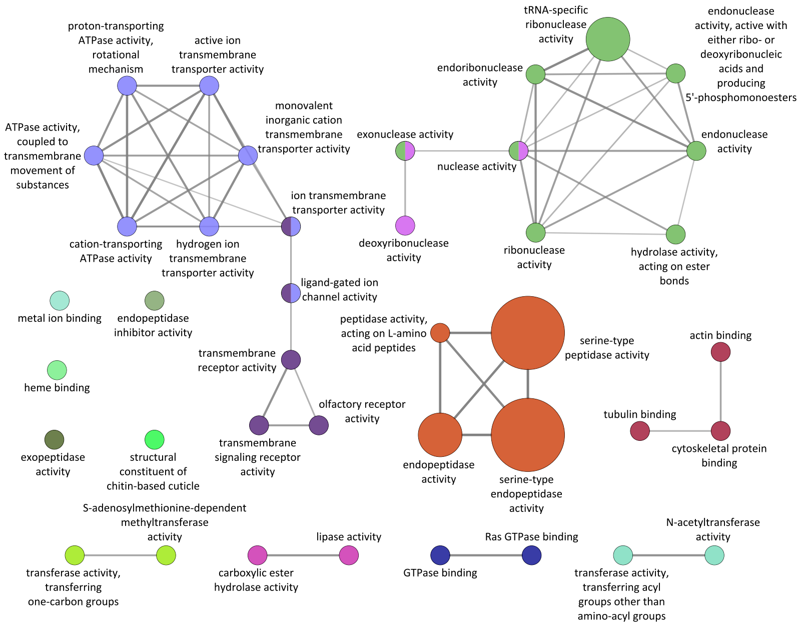

Fig. S18 Network clustering of Molecular Function terms of the TOP10 loci. Network clustering was performed using ClueGo using the following parameters: Min GO Level = 3, Max GO Level = 8, All GO Levels = false, Number of Genes = 3, Get All Genes = false, Min Percentage = 4.0, Get All Percentage = false, GO Fusion = true, GO Group = true, Kappa Score Threshold = 0.4, Over View Term = SmallestPValue, Group By Kappa Statistics = true, Initial Group Size = 1, Sharing Group Percentage = 50.0

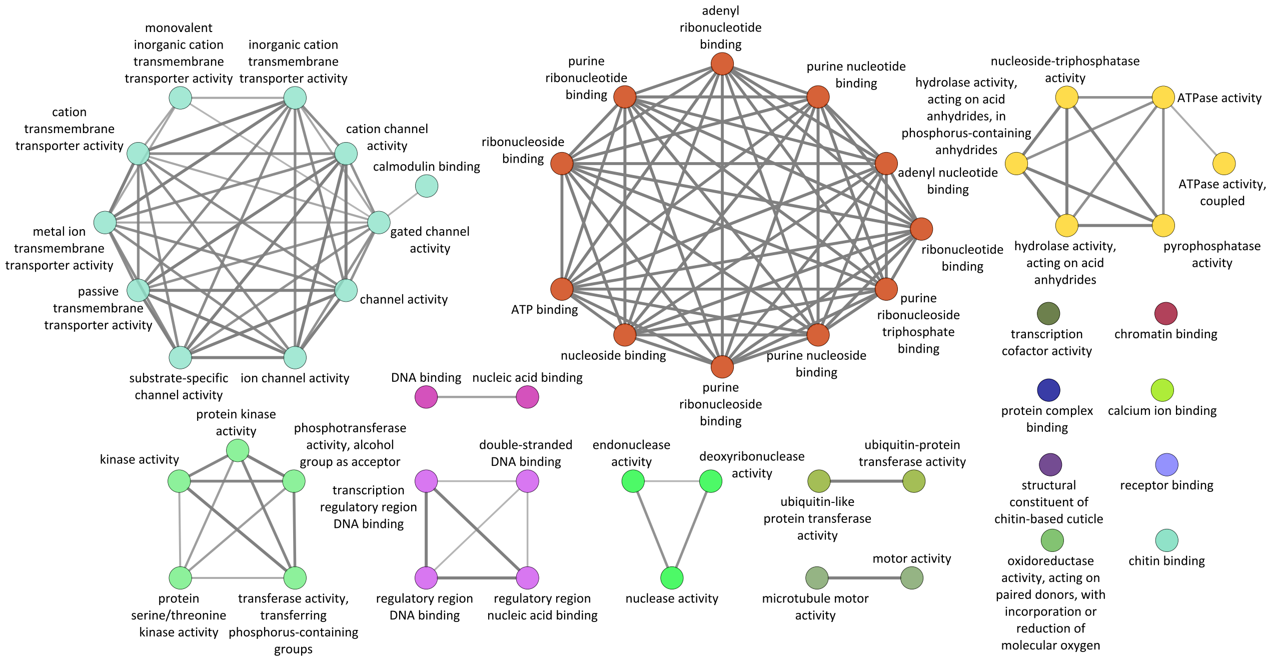

Fig. S19 Network clustering of Molecular Function terms of the positive selected (via PAML with FDR correction) loci. Network clustering was performed using ClueGo using the following parameters: Min GO Level = 3, Max GO Level = 5, All GO Levels = false, Number of Genes = 10, Get All Genes = false, Min Percentage = 8.0, Get All Percentage = false, GO Fusion = false, GO Group = true, Kappa Score Threshold = 0.4, Over View Term = SmallestPValue, Group By Kappa Statistics = true, Initial Group Size = 1, Sharing Group Percentage = 50.0

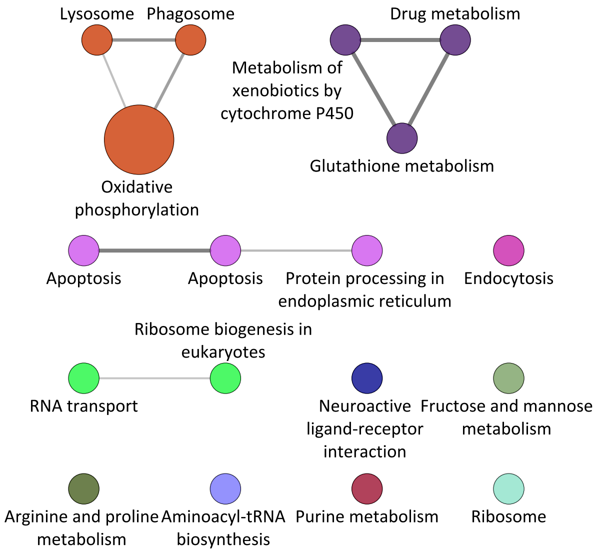

Fig. S20 Network clustering of KEGG terms of the TOP10 loci. Network clustering was performed using ClueGo using the following parameters: Min GO Level = 3, Max GO Level = 8, All GO Levels = false, Number of Genes = 2, Get All Genes = false, Min Percentage = 1.0, Get All Percentage = false, GO Fusion = true, GO Group = true, Kappa Score Threshold = 0.25, Over View Term = SmallestPValue, Group By Kappa Statistics = true, Initial Group Size = 1, Sharing Group Percentage = 50.0

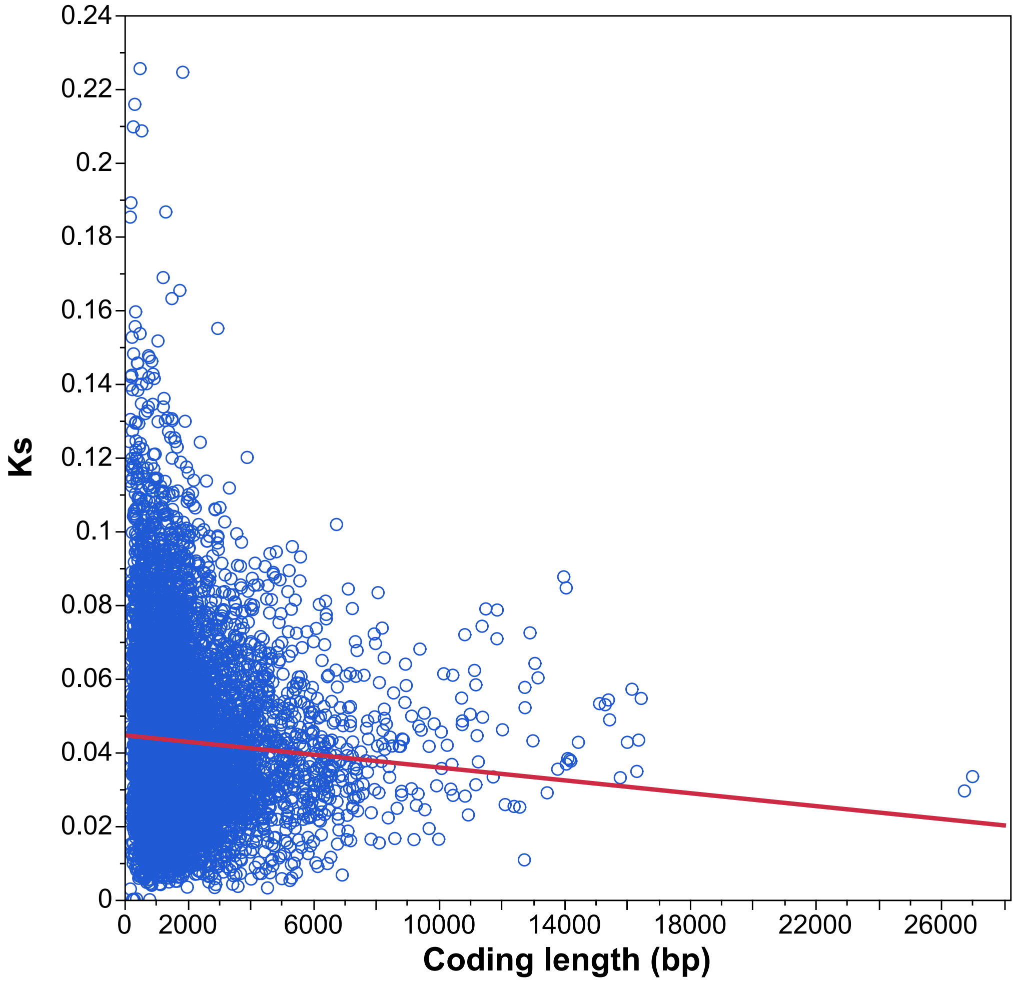

Fig. S21 Relationship between Ks and gene coding length (9,087 loci) ($r^2 = 0.004$, $P < 0.001$).

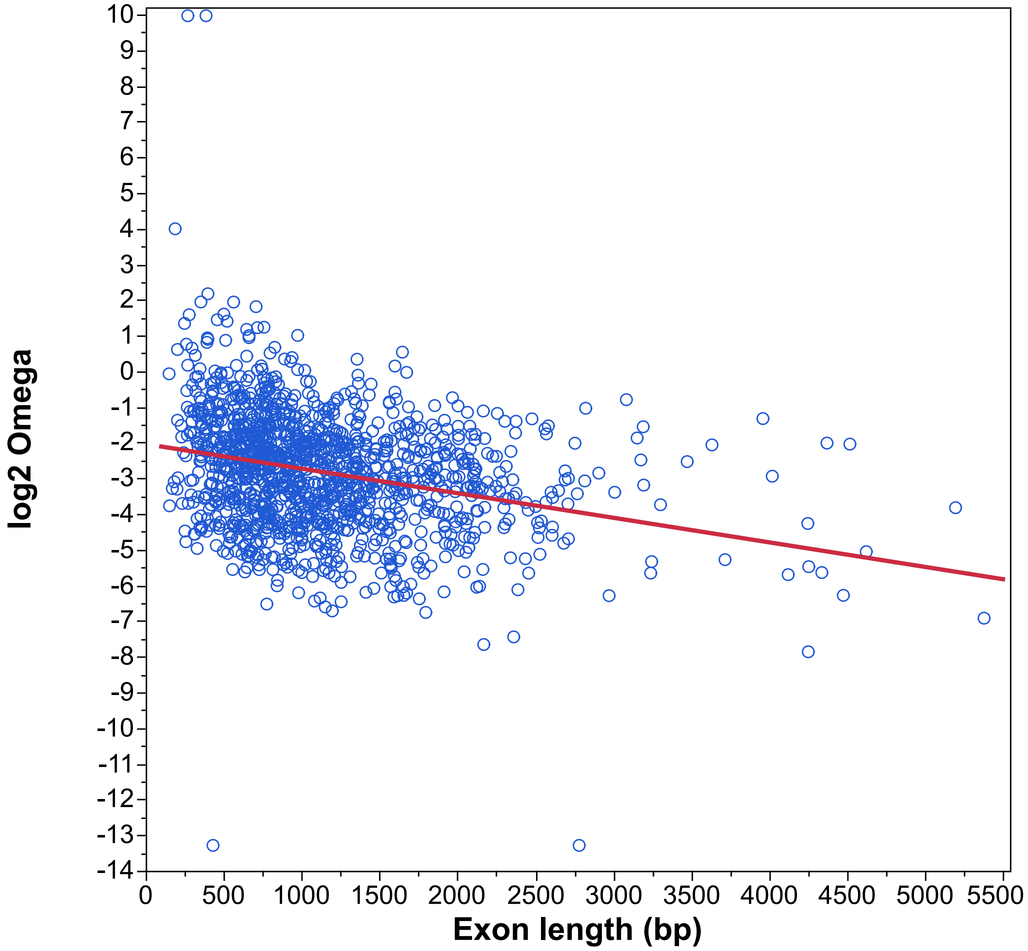

Fig. S22 Relationship between $\log_2 \omega$ and exon length among single exon genes (1,372 loci) (r^2 = 0.08, P < 0.001).

References

1. Delprat A, Etges WJ, Ruiz A. Reanalysis of polytene chromosomes in *Drosophila mojavensis* populations from Santa Catalina Island, California, USA. Drosophila Information Service 2014, 97:53-57.
2. Graveley BR, Brooks AN, Carlson J, Duff MO, Landolin JM, Yang L, Artieri CG, van Baren MJ, Boley N, Booth BW *et al*. The developmental transcriptome of *Drosophila melanogaster*. Nature 2011, 471(7339):473-479.
3. Matzkin LM. Population transcriptomics of cactus host shifts in *Drosophila mojavensis*. Mol Ecol 2012, 21(10):2428-2439.
4. Matzkin LM, Markow TA. Transcriptional differentiation across the four cactus host races of *Drosophila mojavensis*. In: Speciation: Natural Processes, Genetics and Biodiversity. Edited by Michalak P. Hauppauge: Nova Science Publishers Inc.; 2013: 119-136.
